# Physiological and biochemical alterations in *Vigna rdiate* L. triggered by sesame derived elicitors as defense mechanism against *Rhizoctonia* and *Macrophomina* infestation

**DOI:** 10.1038/s41598-023-39660-y

**Published:** 2023-08-24

**Authors:** Kandaswamy Kalaivani, Sengottayan Senthil-Nathan, Vethamonickam Stanley‑Raja, Prabhakaran Vasantha-Srinivasan

**Affiliations:** 1 Post Graduate and Research Centre, Department of Zoology, Sri Parasakthi College for Women, Courtrallam, Tenkasi, Tamil Nadu 627 802 India; 2 Division of Biopesticides and Environmental Toxicology, Sri Paramakalyani Centre for Excellence in Environmental Sciences, Manonmaniam Sundaranar University, Alwarkurichi –Tenkasi, Tamil Nadu 627 412 India; 3 Department of Bioinformatics, Saveetha School of Engineering, Saveetha Institute of Medical and Technical Sciences (SIMATS), Chennai, India

**Keywords:** Chemical biology, Plant sciences, Environmental sciences

## Abstract

Improving agricultural products by the stimulation of plant growth and defense mechanisms by priming with plant extracts is needed to attain sustainability in agriculture. This study focused to consider the possible improvement in *Vigna radiata* L. seed germination rate, plant growth, and protection against the natural stress by increasing the defense mechanisms through the incorporation of *Sesamum indicum* phytochemical compounds with pre-sowing seed treatment technologies. The gas chromatography coupled with mass spectroscopy (GC–MS) analysis revealed that the methanol extract of *S. indicum* leaf extract contained eight major bioactive compounds, namely, 2-ethylacridine (8.24%), tert-butyl (5-isopropyl-2-methylphenoxy) dimethylsilane (13.25%), tris(tert-butyldimethylsilyloxy) arsane (10.66%), 1,1,1,3,5,5,5-heptamethyltrisiloxane (18.50%), acetamide, N-[4-(trimethylsilyl) phenyl (19.97%), 3,3-diisopropoxy-1,1,1,5,5,5-hexamethyltrisiloxane (6.78%), silicic acid, diethyl bis(trimethylsilyl) ester (17.71%) and cylotrisiloxane, hexamethyl-(4.89%). The *V. radiata* seeds were treated with sesame leaf extract seeds at concentrations 0, 10, 25, 50, and 100 mg/L. Sesame leaf extract at 50 and 100 mg/L concentrations was effective in increasing the germination percentage and the fresh and dry weights of roots and shoots. The increased peroxidase activity was noticed after treatment with *S. indicum* extract. In addition, disease percentage (< 60%) of both fungal pathogens (Rhizoctonia and Macrophomina) was significantly reduced in *V. radiata* plants treated with 100 mg/L of sesame leaf extract. These results revealed that physiochemical components present in *S. indicum* mature leaf extract significantly enhanced growth and defense mechanism in green gram plants.

## Introduction

*Vigna radiata* **L.** (green gram or mung bean) is a pulse belonging to the family *Fabaceae* and is one of the most widely cultivated crops in India. It is considered a native species since 1,500 B.C. to India, from where the cultivation spread to Southeast Asia. It grows in a tropical and subtropical warm climate and can tolerate any adverse climatic conditions^1^. This leguminous plant is grown throughout the year, and the pods are consumed as mature and sprouted seeds. It is an economic and rich source of plant-based protein, which enhances the quality of protein in our body. The phytochemical compounds present in the food play an important role in the antioxidant, anti-cancerous, antioxidant, anti-inflammatory, anti-bacterial, and hypolipidemic activities^2^. Plant-based nutrients are a good source of polyphenolic content including flavonoids, tannins, flavones, triterpenoids, steroids, saponins, and alkaloids. *V. radiata* seeds contain phenolic compounds such as caffeic acid, catechin, chlorogenic acid, ferulic acids, gallic acid, vanillic acid, p-coumaric, quercetin and sinapic, syringic acid, 1,2-dihydroxybenzene, 3,4-dihydroxybenzoic acid^3–6^, and flavonoid compounds such as apigenin, naringenin, eriodictyol, kaempferol, quercetin, luteolin, vitexin, and isovitexin^3,5,7^. The bioactive compounds present in the *V. radiata* depend on germination and its growth^8^. The health-promoting bioactive compounds are present in *V. radiata* including peptides, polyphenols, and polysaccharides, therefore, becoming a popular functional food in promoting good health. The mung bean helps to prevent the risk of diabetes mellitus, high blood cholesterol, the severity of obesity, high blood pressure, paralysis, rheumatism, neural disorders, cancer, and melanogenesis, as well as possess hepatoprotective and immunomodulatory activities^5^.

Sesame (*Sesamum indicum* L.) belongs to the family *Pedaliaceae,* and is considered a known domesticated crop in India. The Sesame is a tropical crop that needs warm climatic conditions during its growth period. It is one of the earliest-known crop-based oils in which about 50% of the oil content in seed contains high protein^9^. Sesame is considered “queen of oilseeds” due to the presence of rich polyunsaturated fatty acids (omega-3 and omega-6) and natural phenolic compounds such as lignans, sesamol, and sesaminol^10^. The sesame seed and oil possess nutraceutical, antioxidant, hepatic and cardio protection, cancer and tumor prevention, and antibacterial, antiviral, and anti-inflammatory properties. It is also used as a solvent for intramuscular injections in the pharmaceutical industry and has nourishing, softening and soothing effects. In industries, sesame is used in the production of paints, laxatives, soaps, cosmetics, biodiesel, perfumes, bactericides, and insecticides^11–15^.

Phytochemicals, also known as bioactive compounds, have characteristic physiochemical properties. The leaf extract of *S. radiatum* contains phytochemicals such as alkaloids, flavonoids, polyphenols, coumarins, quinones, tannins, gallic acid, catechins, sterols, terpenoids, saponins, and reducing compounds^16–18^. The phenolic compound accumulated in the sesame leaves in the form of acteoside, isoacteoside, martynoside, verbascoside, plantamajoside, and sesaminol^19^. The bioactive phenolic compounds are associated with plant growth regulation and activation of chemical defense mechanisms^20^. Bioactive components extracted from different parts of the sesame plant possess potential insecticidal, larvicidal, fungicidal, antimicrobial, and antioxidant activities^21–25^.

Among different biotic stresses affecting these crops, fungal pathogens reduce mung bean yield approximately 40–60%^25^. Pathogenic fungi infect *V. radiate* at different growth stages, including seedling, emergence, reproductive, and vegetative stages, resulting in substantial yield loss. Species of the genera Rhizoctonia (wet root rot), Fusarium (wilt), and Macrophomina (dry root rot) infect the mung bean plants during seed emergence/seedling stages. The *Macrophomina phaseolina* commonly known as charcoal rot fungus has a broad host range, and infects the basal stem and the roots^26^. The soil-borne pathogens, especially *M. phaseolina* (Tassi) Goid, *R. solani* (Kiihn), and *Fusarium* spp., attack roots, prevent nutrition uptake, and produce root rot disease complex resulting in the death of plants^25^.

The bioactive compounds in plants act as elicitors and stimulate plant growth, protect plants against pathogens, and induce physiological changes in the plants^27,28^. The plant-derived elicitors play a significant role in enhancing the biosynthesis of secondary metabolites^29^. New alternative research promotes the possibility of using the natural elicitor as a bio-stimulant. The sesame leaf extract proffers an economically feasible and eco-friendly alternative, thus improving seed germination, seedling growth, and defense mechanisms, which promote sustainable agriculture. This study aims to use the sesame leaf extract as a bio-stimulant for the growth promotion in green gram.

## Results

### Identification of sesame leaf extract phytochemicals by gas chromatography coupled with mass spectroscopy (GC–MS)

The results demonstrated that the methanolic extract of sesame leaves exhibited a total yield percentage was 45.8% volume. The components present in the methanol leaf extract of the sesame plant (*S. indicum)* were identified by gas chromatography coupled with mass spectroscopy (GC–MS) analysis, and eight phytoconstituents were identified (Table 1). The compounds identified were 2-ethylacridine (8.24%), tert-butyl (5-isopropyl-2-methylphenoxy) dimethylsilane (13.25%), tris(tert-butyldimethylsilyloxy) arsane (10.66%), 1,1,1,3,5,5,5-heptamethyltrisiloxane (18.50%), acetamide, N-[4-(trimethylsilyl) phenyl (19.97%), 3,3-diisopropoxy-1,1,1,5,5,5-hexamethyltrisiloxane (6.78%), silicic acid, diethyl bis(trimethylsilyl) ester (17.71%), cyclotrisiloxane, and hexamethyl-(4.89%). The known spectrum of GC–MS in the methanol extract was found in phytochemical components with retention times 16.828 min, 16.865 min, 16.969 min, 17.026 min, 17.263 min, 17.329 min, 17.386 min, and 21.792 min respectively (Table 1).

**Table 1:** Chemical composition of leaf extract of *S. indicum.*

Peak no	RT	Peak area %	Compound name	Molecular formula	Molecular weight	Chemical structure
1	16.828	8.24	2-Ethylacridine	C1_5_H1_3_N	207.27	
2	16.865	13.25	tert-Butyl(5-isopropyl-2-methylphenoxy)dimethylsilane	C1_6_H_28_OSi	264.48	
3	16.969	10.66	Tris(tert-butyldimethylsilyloxy)arsane	C_18_H_45_AsO_3_Si_3_	468.7	
4	17.026	18.50	1,1,1,3,5,5,5-Heptamethyltrisiloxane	C_7_H_21_O_2_Si_3_	221.5	
5	17.263	19.97	Acetamide, N-[4-(trimethylsilyl)phenyl	C_11_H_17_NOSi	207.34	
6	17.329	6.78	3,3-Diisopropoxy-1,1,1,5,5,5-hexamethyltrisiloxane	C_12_H_32_O_4_Si_3_	324.63	
7	17.386	17.71	Silicic acid, diethyl bis(trimethylsilyl) ester	C_10_H_28_O_4_Si_3_	296.58	
8	21.792	4.89	Cyclotrisiloxane, hexamethyl-	C_27_H_44_O_3_Si_3_	500.9	

### Seed germination of V. radiata after treatment with S. indicum

**Figure 1:**
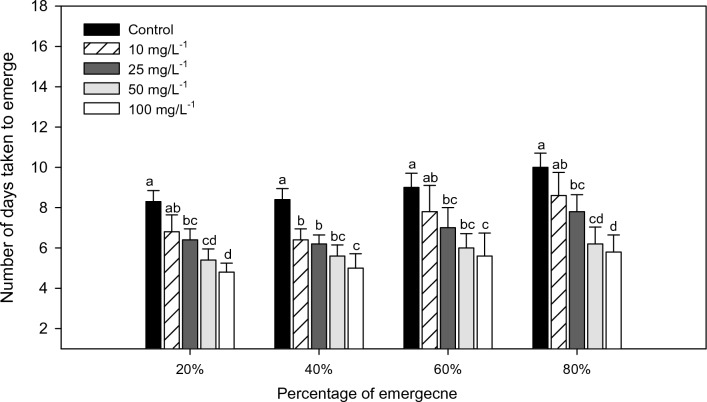
Days until 20%, 40%, 60% and 80% emergence of *V. radiata* under greenhouse condition after seed treatment with *S. indicum* extract. Mean (± SEM) followed by the same letter in an individual experiment indicates no significant difference (*P* < 0.05) in a Tukey’s test (Treatment concentration in mg/L).

The seeds pretreated with different concentrations of sesame leaf extract (10, 25, 50, 100 mg/L) showed enhanced levels of germination percentage as compared to the control. The germination percentage increased proportionately with an increased concentration of sesame leaf extract (Fig. 1). The *V. radiata* seeds treated with sesame extract displayed increased emergence compared with the control plant. In *V. radiata*, germination percentages of 80%, 60%, 40%, and 20% emergence occurred analogously with the increasing concentrations from 10, 25, 50, 100 mg/L (80%—*F*_*4,20*_ = 19.18; *P* < 0.0001, 60% *F*_*4,20*_ = 9.46; *P* < 0.0001, 40%—*F*_*4,20*_ = 25.81; *P* < 0.0001 and 20%- *F*_*4,20*_ = 17.22; *P* < 0.000) respectively.

### Root and shoot length of V. radiata after treatment with S. indicum

**Figure 2:**
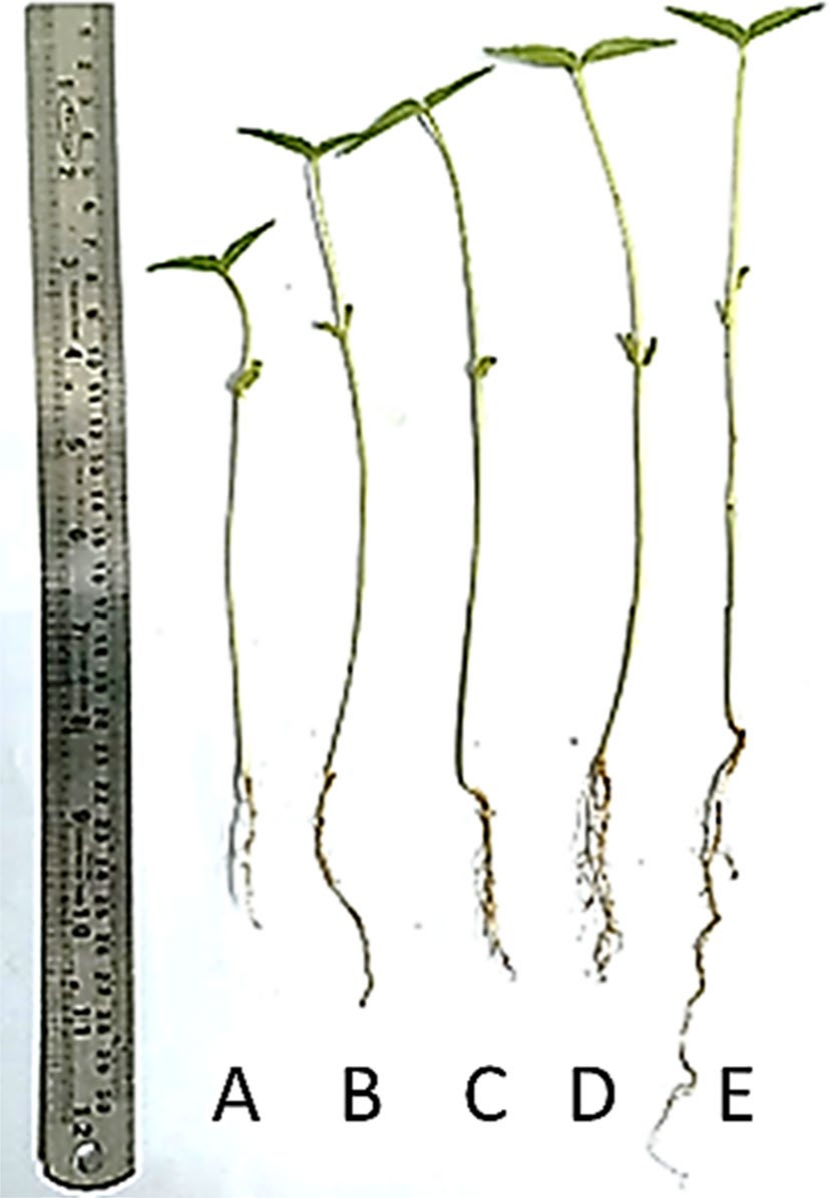
External morphology of *V. radiata* 11 days post treatment with *S. indicum* extract (**A** Control; **B** 10 mg/L, **C** 25 mg/L, **D** 50 mg/L, and **E** 100 mg/L).

**Figure 3:**
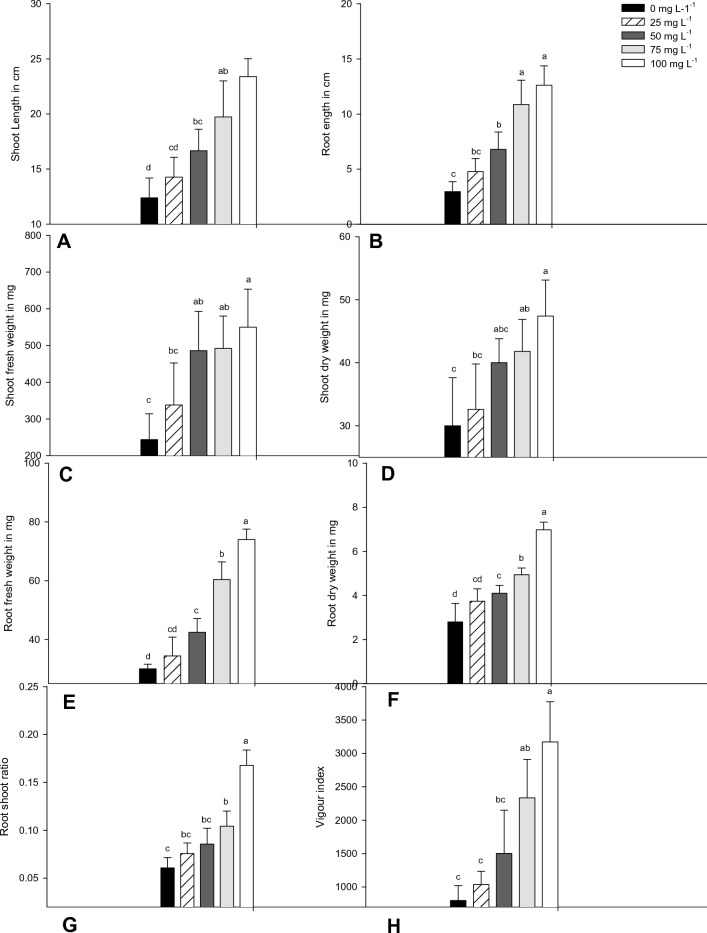
Shoot and root length in cm (**A**, **B**), shoot fresh and dry weight in mg (**C**, **D**), root fresh and dry weight in mg (**E**, **F**), root to shoot ratio and seed vigour (**G**, **H**) of *V. radiata* 11 days post treatment with *S. indicum* extract. Mean (± SEM) followed by the same letter in an individual experiment indicates no significant difference (*P* < 0.05) in a Tukey’s test (Treatment concentration in mg/L).

The root and shoot length of leaf extract primed *V. radiata* seeds is represented in figure. After five days of germination, the result showed that sesame leaf extract significantly increased the root length and shoot length compared with the control seeds (Fig. 2). Seed priming treatment enhanced the seedling growth with increased effect from 50 mg/L and maximum at 100 mg/L leaf extract. On average, seed priming treatment effectively enhanced the shoot length up to 52.17% after treatment with 100 mg/L (*F*_4,20_ = 20.27, *P* < 0.0001) and root length up to 77%* F*_4,20_ = 32.62, *P* < 0.0001) (Fig. 3A, B).

### Root and shoot fresh weight of V. radiata after treatment with S. indicum

Figure 3C–F indicates the efficacy of sesame leaf extract treatment in enhancing the root and shoot fresh weight. The maximum root and shoot fresh weight were recorded in 100 mg/L treated seeds, wherein root and shoot fresh weight of 78 mg and 680 mg (*F*_*4,20*_ = 75.16; *P* < 0.0001 and *F*_*4,20*_ = 8.38; *P* < 0.0001) were recorded. Similar trends were observed in the root and shoot dry weights (*F*_*4,20*_ = 42.45; *P* < 0.0001). However, the maximum root and shoot fresh weights of 50 mg/L, 25 mg/L, and 10 mg/L concentrations, and the control were 66 mg, 48 mg, 42 mg, 32 mg, and 620 mg, 580 mg, 480 mg, and 320 mg respectively. Further weight gain was observed in fresh and dry weight of shoot root after leaf extract priming of *V. radiata*. Overall, a positive relationship was observed between the concentrations of the leaf extract with rate, shoot, root fresh, and dry weights (Fig. 3C–F).

### Root to shoot ratio of V. radiata after treatment with S. indicum

The root to shoot ratio depends upon the accumulation of sesame leaf extract in seed treatments, which influences the stimulation of root and shoot growth. The seed treatment with increasing concentration of sesame leaf extract increased the root to shoot ratio from 0.0758 of 10 mg/L to 0.1678 of 100 mg/L, as against the control with a ratio of 0.06080 (*F*_*4,20*_ = 21.67; *P* < 0.0001) (Fig. 3G).

### Seed vigor index of V. radiata after treatment with S. indicum

The effect of different concentrations of sesame leaf extract on germination percentage, root, and shoot length was determined by the evaluation of seed vigor index. The highest vigor index of 3172 100 mg/L was observed when treated with 100 mg/mL, followed by 2334, 1503, 1034.4, and 798 for the concentrations 50 mg/L, 25 mg/L, 10 mg/L, and control, respectively (*F*_4,20_ = 18.07, *P* < 0.0001). The increase in the concentration of the leaf extract enhanced the growth parameters under consideration proportionately (Fig. 3H).

### Peroxidase activity of V. radiata after treatment with S. indicum

**Figure 4:**
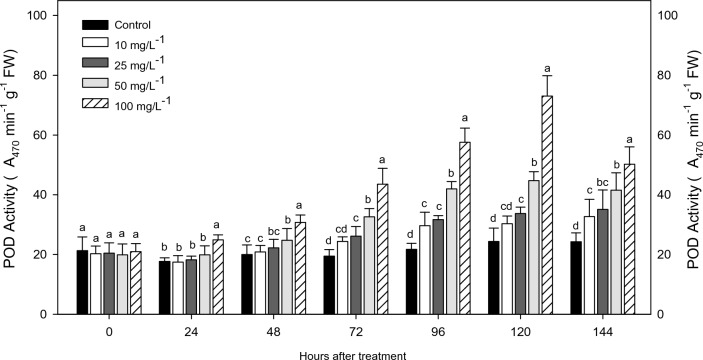
Effects of *S. indicum* extract treatments on POD activity in *V. radiata* plant. Mean standard error ( ±) followed by the same letter within bar indicates no significant difference (*P* ≤ 0.05) in a Tukey test.

The *V. radiata* treated leaves were assessed for alterations in peroxidase (POD) enzyme activity. The leaves exhibited increased POD activity when treated with 100 mg/L concentration of the sesame leaf extract (*F*_4,20_ = 13.13; *P* < 0.0001). The POD activity in leaves was found to be 117.9 min^−1^ g^−1^FW when treated with 100 mg/L of the leaf extract. In contrast, the plants treated with 10 mg/l of the leaves extract exhibited POD activity of 79.27 min^−1^ gv^−1^FW, which was higher than that of the control (76.24 min^−1^ g^−1^ FW). The POD activities at different concentrations of sesame leaf extract were significantly accelerated at higher concentration (Fig. 4).

The increasing peroxidase activity was recorded from 24 to 144 h on control and leaf extract primed *V. radiata* plants at 0 h (*F*_4,20_ = 1.06; *P* < 0.988-not significant) and 24 h (*F*_4,20_ = 3.98; *P* < 0.006). However, the enzymatic responses were insignificant in all treatments except 100 mg/L. A stable and increased level of POD activity higher than the control was observed after 24 h. As against the control, the treated plants displayed a seven-fold increase in POD activity.

### Incidence of fungal pathogens on mung bean

**Figure 5:**
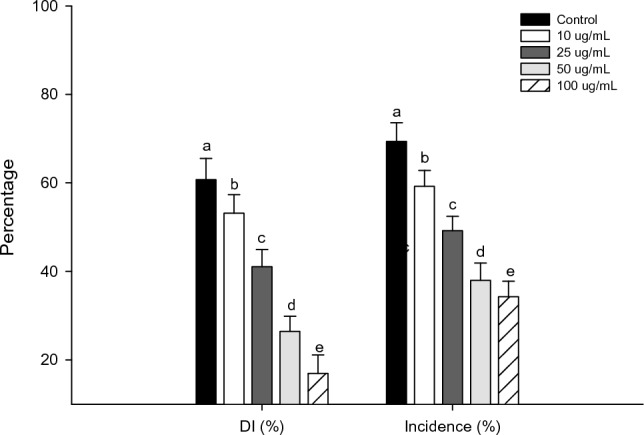
Efficacy of *S. indicum* extract in control of bacterial blight disease on rice leaves of *X. oryzae* (DI- Disease index and Incidence percentage). Mean standard error ( ±) followed by the same letter within bar indicates no significant difference (*P* ≤ 0.05) in a Tukey test.

**Figure 6:**
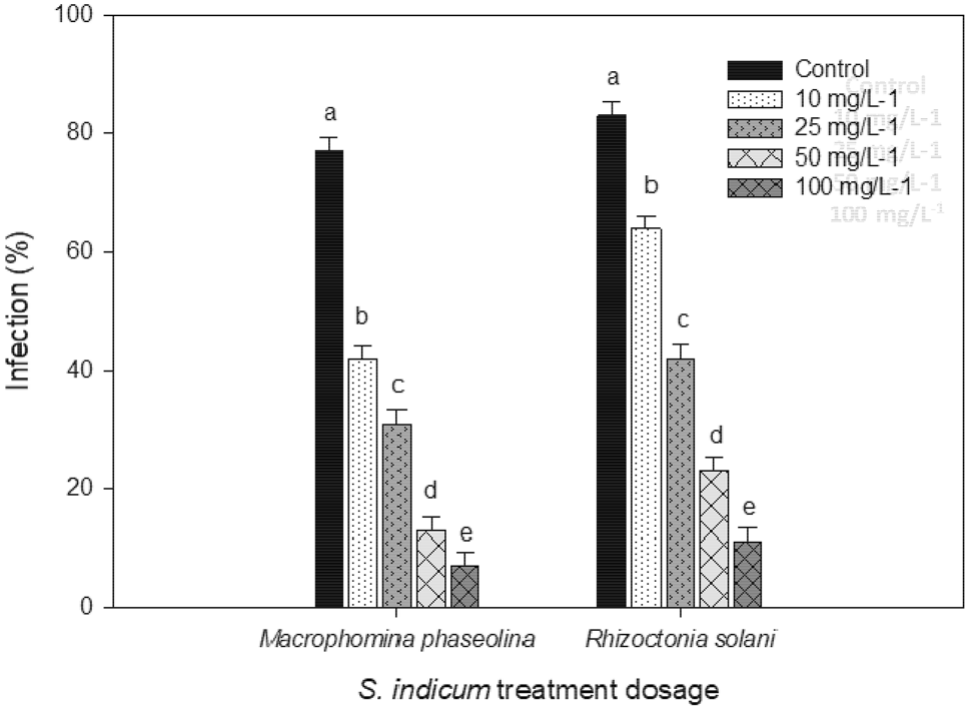
Incidence of root rot fungi *M. phaseolina* on mung bean post treatment with *S. indicum*. Mean standard error ( ±) followed by the same letter within bar indicates no significant difference (*P* ≤ 0.05) in a Tukey test.

Soil amendment with *S. indicum* extracts showed significant suppression of the root rot fungi, *M. phaseolina* and *R. solani.* The reduction of fungal infection was maximum (*P* < 0.001) on mung bean when the soil was amended with *S. indicum* 100 mg/L (Fig. 5). Also, all the treatment dosages (10 mg/L, 25 mg/L, 50 mg/L, and 100 mg/L) were higher than the control in both *M. phaseolina* (*F*_4,20_ = 12.23; *P* < 0.0001) and *R. solani* (*F*_4,20_ = 16.73; *P* < 0.0001) (Fig. 6).

## Discussion

Seed treatments with *S. indicum* extract increased germination percentage growth in *V. radiata* in a dose-dependent manner. Also, the stimulatory effects appeared more pronounced at 75 and 100 mg/L seed treatments. The plant extract acts as a bio-stimulant, accelerating crop productivity by enhancing germination, growth, and biomass and activating defense-related enzymes in plants^30^.In this study, sesame leaf extract was used as a bio-stimulant and as an elicitor to improve green gram production. The methanol extract of *S. indicum* L. leaves was screened by gas chromatography-mass spectrometry (GC–MS) analysis for qualitative phytochemical detection. The spectrum revealed the presence of eight major phytochemical compounds. In a similar study, 2-ethylacridine, silicic acid, and diethyl bis (trimethylsilyl) were identified by the mass spectrometric analysis of the *Dicranopteris linearis* stem extract, and the identified phytochemicals possessed significant antimicrobial and antioxidant potential^31^. The methanolic extract from the leaves of *Centella asiatica* contained the bioactive compound tert-Butyl (5-isopropyl-2-methylphenoxy) dimethylsilane that had antimicrobial properties^32^. The ethanolic extracts of *Olea europaea* isolates showed the presence of phenolic compounds tris (tert-butyldimethylsilyloxy) arsane, silicic acid, diethyl bis(trimethylsilyl) ester, and cyclotrisiloxane hexamethyl in the latex of the plants with effective antimicrobial properties^33^. The bioactive compound 1,1,1,3,5,5,5-Heptamethyltrisiloxane was extracted from *Allium atroviolaceum* flowers that exhibited antibacterial activity against *Bacillus subtilis* and enhanced the photosynthetic capacity of the plants^34^. Ethyl acetate extract consisted 3,3-diisopropoxy-1,1,1,5,5,5-hexamethyltrisiloxane, cyclotrisiloxane, and hexamethyl isolated from the bark of *Trichilia gilgian*. The phytochemicals were inhibitory against the *Sitophilus zeamaïs and Rhyzopertha dominica* at high doses of the extract (1 g/10 L^−1^ and 0.5 g/10 L^−1^)*.* The fruit extract of *Salvadora persica L*. was found to possess 3,3-diisopropoxy-1,1,1,5,5,5-hexamethyltrisiloxane and cyclotrisiloxane hexamethyl compound that had antimicrobial activity against *Streptococcus mutans*^35^. The leaves and bark extracts derived from *Dillenia scabrella* contained the phytochemicals cyclotrisiloxane hexamethyl, silicic acid, and diethyl bis (trimethylsilyl) ester, which displayed antioxidant and anti-diabetic activities^36^. Acetone crude extract of *Turbinaria decurrens* seaweeds comprised cyclotrisiloxane hexamethyl with potential antimicrobial, antioxidant, and antibacterial properties^37^.

*S. indicum* is a valuable oil seed plant known to be relatively rich in bioactive compounds. The results showed that seed treatment with sesame leaf extract have significant impact on *V. radiata* germination and plant growth. The bioactive compounds present in the extract improve the agricultural yield by pretreating the seeds^38,39^.

The sesame leaf extract-treated seeds significantly enhanced the percentage of germination when compared with untreated seeds. *V. radiata* seeds treated with sesame leaf extract (100 mg/L) showed a considerable increase in the percentage of germination when compared with the lower concentrations and the control. In an independent study, phenolic compounds were extracted from grape seeds and spruce bark, which showed a significant stimulatory effect on the radicle length of *Phaseouls vulgaris* seeds^40^. The seed treatment with an oil-based solution of tyrosol (10 mg/L) extracted from *Phomopsis sp.* displayed improved germination percentage of soybean plant^41^. The extract obtained from the seaweed, *Laurencia obtuse,* induced maximum seed germination at a concentration of 20 g/L^42^.

The root and shoot length varied significantly when compared to that of the untreated seed. The sesame leaf extract treated seeds showed significant increase in root and shoot length (12.6 cm and 23.4 cm in 100 mg/L) than that of control (2.9 cm and 12.4 cm). However, the root and the shoot lengths were comparable when treated with lower concentrations of the leaf extract and in the control. The bioactive compounds act as stimulators and have an important role in enhancement of the growth and development of the plants^43^. Application of trehalose (Tre) and salicylic acid (SA) on drought stress sweet basil (*Ocimum basilicum* L.) plant showed enhanced plant growth by increasing the root and shoot length^44^. The spruce bark, *Asclepias syriaca* and grape seeds extracts stimulated radicle elongation in *Phaseouls vulgaris* than the control solution^40^.

The effect of various concentrations of sesame leaf extracts in seed treatment could be seen in the vigor index. The increased vigor index was noticed in seeds treated with a higher concentration of sesame leaf extracts 100 mg/L and followed by 50 mg/ L, 25 mg/L, 10 mg/L, and control, whereas the decreased vigor index was recorded in 10 mg/L and control. At the concentrations of 25 mg/L and 50 mg/L, the leaf extract of *Azadirachta indica* and bark of *Boswellia dalzielii* extract significantly enhanced the germination rate and vigor index of cotton seeds^45^.

The result reported that sesame leaf extract of 100 mg/L had increased significantly in the growth of root and shoot fresh (74 mg and 550 mg) and dry weights (6.98 mg and 47.40 mg) than that of control fresh (30 mg and 244 mg) and dry weights (2.8 mg and 30 mg). Similarly, seed-priming of carrots with 50 and 100 mg/L with alpha-tocopherol (Toc) showed a significant level of plant biomass (*p* ≤ 0.001and 0.01) accumulation when exposed to drought conditions^46^. The polyphenolic extracts facilitated the assimilation of the fresh biomass in *Phaseouls vulgaris* germinated seeds^40^.

Seed soaking with sesame leaf extract induced maximum increase in root shoot ratio while an increase in concentration was observed by 10, 25, 50, and 100 mg/L seed treatment.

The sesame leaf extract induced the peroxidase (POD) enzyme activity, which has a key role in stimulating the plants defense mechanism. POD activity increased significantly (*p* ≤ 0.001) in carrot cultivars under normal and drought conditions^46^. Seed treatment with methyl salicylate (MeSA) at 50 and 100 mg/L concentrations significantly increased POD activity in IR 20, IR 50, IR 64, ASD 16, ASD 19, and ADT 46 rice (*Oryza sativa* L) varieties^20^. Phenolic compounds and its derivatives have been used to accelerate the defense mechanism against the pathogen attacks^38^.

## Conclusion

Plant based material is a key source of bioactive compounds. The result clearly focus on the potential of bioactive compounds derived from the leaf extract to improve crop yield. Thus, seed treatment with methanolic sesame leaf extract effectively increased all the growth parameters under consideration, and also accelerated POD activity in plant. Further, we conclude that use of *S. indicum* in seed treatment has excellent potential for increasing ideal emergence and biomass accumulation, thus produce promising effects on the growth of *V. radiata.*

## Materials and methods

### Collection and preparation of leaf sample

This study was carried out in July to October 2021and the location of the experiment had coordinates latitude of 8.946481° N and longitude of 77.289979° E. During the experimental period, the climatic conditions were 22–32 °C day/night temperature fluctuation, 12–13 h photoperiod and 81–83% relative humidity (RH) during the day. The leaves of *S. indicum* were collected from the field, Tenkasi, Tamil Nadu, India*.* Fresh leaves were collected from the matured plant during the ripening phase. The leaves were cleaned and shade dried for a period of 10 days at room temperature 28 ± 3 °C. The dried leaves were then powdered using a mixer grinder. Cultivars mentioned above were used for research purpose only and it does not come under endangered species of wild flora and fauna as per IUCN. Essential methods and guidelines are adopted from the IUCN.

### Preparation of methanol plant extracts of sesame leaves

The powdered leaves (100 g) were extracted using a magnetic stirrer with methanol 500 L^−1^ for 48 h until the solvent was extracted as a clear substance. The extracts were filtered using Whatman filter paper no. 1, and the solvents were evaporated using a water bath at 60 °C to yield the crude extract. The yield of fine slurry extracts was 45.8% volume by the weight of the extract after evaporation of solvent and the weight of the plant powder. The plant extracts were stored in bottles for the further experiments^47^. The crude plant extracts (200 mg/ml) were further dissolved in 2 ml of 1% dimethyl sulfoxide (DMSO) to get standard stock solutions. The different concentrations were prepared by using the stock solutions (10, 25, 50, and 100 mg/L).

### Phytochemical analysis

The phytochemical analysis of the *S. radiatum* leaf extract was carried out to find the phytochemicals like alkaloids, saponins, flavonoids, terpenoids, tannins, steroids, quinones, and phenols by using the standard method.

### Collection of seed and seed preparation

*V. radiata* seeds (green seeds) were primed with different concentrations of leaf extract. The seeds were obtained from the nearby agricultural store in Nannagaram, Courtallam, Tenkasi, Tamil Nadu, India with latitude 8.9449° N and longitude 77.2930° E. Seeds were selected with uniform size. To evaluate the effects of sesame leaf extract on seed germination, a petri plate experiment, and seedling vigor evaluation were conducted in the laboratory. For the experiment purpose, the seeds were surface sterilized using 2% mercuric chloride for 2 min and the seeds were rinsed repeatedly with distilled water. Seeds were soaked in prepared concentrations of plant extract as a test solution and water as a control for 5 h.

### Seed germination test and seedling measurement of V. radiata after treatment with S. indicum

A total of 50 seeds were placed on the petri dish for germination. The seeds were treated with different concentrations of plant extract, and the control seeds were treated with distilled water. After a period of 5 h, each petri dish was covered with a lid, and observed for seedling emergence and germination at regular intervals for five days^48^. For the statistical analysis, the percentage of germination was recorded once the formation of radicle length had reached 2 mm^49^.

### Seedling vigour of V. radiata after treatment with S. indicum

The treated and control seeds were sown at a depth of 3 cm in plastic bags of 8 inches in length and 6 inches in width, containing soil with a mixture of two parts of silt loam soil and one part of sand. The plants were kept in greenhouse conditions and watered regularly. The seedling growth was recorded on the 11th day from the start of the experiment. The root and shoot lengths were recorded in centimeters using a ruler. Each treatment contained five replications, and for each replication, five seeds were used.

### Seedling length and weight of V. radiata after treatment with S. indicum

The root and shoot lengths were measured by selecting five seeds randomly for each treatment. The shoot length was measured by a cut from the base of the hypocotyl, and root length was measured from the tip of the primary root to the base of the hypocotyl. The fresh and dry weights were weighed using an electric balance and expressed in grams for each replicate^27^. The shoot and root weights were examined using monopan balance. The seedling vigor percentage index was calculated by multiplying the germination rate with the seedling length. The root-to-shoot ratio was calculated by the dry weight of the root divided by the dry weight of the shoot for each treatment.

### POD activity of V. radiata after treatment with S. indicum

*S. radiatum* treated *V. radiata* and control seeds were assayed alterations in the peroxidase (POD) activity. The collected leaves were homogenized and centrifuged at 10,000 rpm for 20 min at 4 °C. The supernatant was collected and used the determination of the enzyme activity. The leaves of *V. radiata* were extracted using the guaiacol method^50^. The mixture consisted of 12 mM of 0.01% H_2_O_2_, 50 mM of 0.1 M phosphate buffer (pH 5.8), and 7.2 mM of guaiacol containing a volume of 3L. The absorbance was measured continuously at 470 nm by a spectrophotometer at 25 °C. The phosphate buffer was used as a control. The results were determined by measuring the absorbance of tetra-guaiacol at 470 nm for 3 min at 30 s intervals. POD activity was expressed as unit per minute per gram fresh weight (min^−1^ g^−1^ Fresh Weight).

### Determination of root infecting fungi

Different dosages (10, 25, 50, and 100 ug/mL) of the sesame extract were used for soil amendment. Surface sterilized seeds were sown in 8 cm diameter plastic pots, each containing 300 g soil, and watered regularly to maintain sufficient moisture required for the growth of *V. radiata* plants. The pots were kept in a screen house in a randomized complete block design with three replicates per treatment; seeds treated with sterile distilled water served as control. To determine the incidence of root rot fungi *M. phaseolina* (Gen Bank Accession No. MG434668) and *R. solani* (Gen Bank Accession No. KF312465), 1 cm long root pieces were washed in running tap water and surface sterilized with 1% Ca(OCl)_2_. The pieces were then transferred to PDA plates supplemented with penicillin (200 mg/L) and streptomycin (200 mg/L). The petri dishes were incubated at room temperature for one week, and the progress of the fungal infection on the roots was recorded.

### Statistical analysis

The effect of *S. radiatum* treatment on peroxidase activity with different concentrations was subjected to two-way factorial ANOVA, in which time and concentrations were considered variables. All other data were subjected to one-way ANOVA, and the treatment means were compared by Tukey-family error test (*P* < 0.05) by using the Minitab®17 software package. The data on above said experiments were arcsine transformed (except the percentage of emergence) before undergoing statistical analysis.

## Data Availability

The datasets used in the current study are available with the corresponding author on request.
